# Urban inoculation and the decline of smallpox mortality in eighteenth‐century cities—a reply to Razzell†

**DOI:** 10.1111/ehr.12112

**Published:** 2015-06-15

**Authors:** Romola J. Davenport, Jeremy Boulton, Leonard Schwarz

**Affiliations:** ^1^ University of Cambridge; ^2^ University of Newcastle; ^3^ University of Birmingham

## Abstract

Smallpox was probably the single most lethal disease in eighteenth‐century Britain but was reduced to a minor cause of death by the mid‐nineteenth century due to vaccination programmes post‐1798. While the success of vaccination is unquestionable, it remains disputed to what extent the prophylactic precursor of vaccination, inoculation, reduced smallpox mortality in the eighteenth century. Smallpox was most lethal in urban populations, but most researchers have judged inoculation to have been unpopular in large towns. Recently, however, Razzell argued that inoculation significantly reduced smallpox mortality of adults and older children in London in the last third of the eighteenth century. This article uses demographic evidence from London and Manchester to confirm previous findings of a sudden fall in adult smallpox mortality and a rise in the importance of smallpox in early childhood *c*. 1770. The nature of these changes is consistent with an increase in smallpox transmission in London and Manchester after 1770 and indicates that smallpox inoculation was insufficient to reduce smallpox mortality in large towns. It remains unclear whether inoculation could have operated to enhance smallpox transmission or whether changes in the properties of the smallpox virus drove the intensification of smallpox mortality among young children post‐1770.

Smallpox was the greatest epidemic scourge of eighteenth‐century Europe, and accounted for 10–20 per cent of burials in urban populations in Britain.^1^ There was no effective treatment for the disease but two forms of prophylaxis, inoculation and vaccination, were introduced into Britain over the course of the eighteenth century. Inoculation involved deliberate infection with an attenuated form of smallpox that induced a (usually) mild case of the disease sufficient to confer permanent immunity. Its introduction to England is attributed to Lady Wortley Montagu in the 1720s, but it was used only relatively rarely before the 1760s when a simpler and cheaper method of inoculation became available.^2^ The alternative, vaccination, involved deliberate infection with cowpox, a relatively benign virus that was sufficiently similar to smallpox to induce long‐lasting immunity against smallpox. Vaccination was developed by William Jenner in the mid‐1790s and was adopted very widely following the publication of Jenner's work on cowpox in 1798.^3^ Vaccination was extremely successful in reducing smallpox mortality, and smallpox had become a minor cause of death in Britain by the mid‐nineteenth century. Most of the dramatic decline in smallpox mortality after 1800 is attributed to vaccination rather than inoculation, although inoculation continued to be practised alongside vaccination until it was banned in 1840. What does remain in dispute is whether inoculation significantly reduced smallpox mortality *before* vaccination. Life expectancy improved in the English population over the late eighteenth century, and this trend was most marked in urban areas where smallpox mortality was highest.^4^ Among London Quakers, falls in smallpox mortality accounted for most of the improvements in survival at childhood ages two to 10 years that occurred after 1750, and these falls were probably a consequence of inoculation.^5^ Unfortunately the difficulties of studying highly mobile and rapidly growing urban populations have meant that urban mortality levels and trends remain poorly characterized in this period, and the wider contribution of inoculation to falls in urban mortality before 1800 remains unclear.

With the exception of recent work by Razzell, previous research on the history of inoculation in Britain has been almost unanimous in concluding that the widespread adoption of inoculation was confined largely to rural villages and small towns in eighteenth‐century England.^6^ The popularity of inoculation in smaller settlements and its neglect in large urban populations has been explained as due to several factors. Because inoculation involved deliberate infection with smallpox it carried the risk of spreading the infection from the inoculated person to other susceptible persons. To avoid this possibility, parishes often conducted ‘general inoculations’ in which all susceptible inhabitants of the parish were inoculated simultaneously. This conferred widespread immunity when an epidemic threatened, and protected the population from the risk of an outbreak caused by the inoculation itself. General inoculations appear to have been carried out particularly when a smallpox epidemic threatened, and were usually conducted at parish expense.^7^ However, in large urban centres it was not feasible to inoculate the entire vulnerable population at once. Fears regarding accidental infection by inoculated individuals constituted a major source of contemporary conflict over the use of ‘partial’ inoculation in large towns, and a number of prominent inoculators including Thomas Dimsdale and, initially, William Haygarth were opposed to the ‘promiscuous’ inoculation of urban populations.^8^ A related reason for the popularity of inoculation in villages and small towns is that in these types of parishes mass inoculations made it possible to avert the costs of caring for and burying smallpox victims as well as the adverse consequences of an outbreak for the economy of a market town.^9^ In contrast smallpox was endemic in English cities and formed a constant backdrop to commercial activities. There was little attempt by urban parishes to isolate those infected with smallpox, and this reduced the cost of caring for smallpox victims.^10^ Moreover, the large numbers of susceptible infants born into the population each year and the frequent or constant threat of smallpox infection would have required very frequent inoculations at considerable expense to any parish considering such measures. Therefore the financial motive for mass inoculation that existed in smaller settlements was largely absent in large towns. A third reason given for the apparently low rates of inoculation in urban areas was a certain fatalism on the part of urban parents. By the late eighteenth century smallpox in towns was a disease of children and recent migrants, and it has been argued that many parents regarded smallpox as an inevitable childhood rite of passage.^11^ A number of facilities were established to inoculate urban residents in the late eighteenth century but these were generally considered unsuccessful.^12^ Conversely, it appears that vaccination was adopted subsequently with greater enthusiasm in large towns than in the country where it faced greater competition from inoculation.^13^

The extent and success of inoculation in urban or rural areas is difficult to gauge for several reasons. In addition to the apparently geographically specific nature of inoculation practice (which makes generalizations difficult), much of the explicit evidence regarding inoculation is anecdotal and therefore difficult to quantify. Moreover, the heat of the contemporary debate surrounding the practice of inoculation means that much of the evidence is partisan in nature.^14^ In addition, it is difficult to assess the efficacy of a preventative measure that required repeated application to the constant stream of unprotected infants born into the population, in the absence of routine information on the frequency and coverage of inoculation efforts.

Recently we presented demographic evidence from two London parishes that indicated that a sudden reduction in the incidence of smallpox deaths among adults occurred in London in the 1770s.^15^ Before *c.* 1770 approximately 20 per cent of smallpox burials in London were adults, despite evidence that smallpox was endemic in London and was a disease of childhood among the London‐born population. Our evidence indicated that adult victims in this period were mainly migrants from relatively remote rural areas where smallpox epidemics still remained rare.^16^ However, after 1775 only 5–10 per cent of smallpox victims were adults. We argued that this sudden decline in the risk of smallpox to adult migrants could be explained to some extent by the spread of inoculation against smallpox in rural areas, but may also have resulted from a sudden rise in the infectiousness of the smallpox virus that increased exposure to smallpox within the English population. We were led to this rather surprising conclusion by the evidence of simultaneous changes in patterns of smallpox mortality among children, most of whom would have been London‐born. The apparently abrupt drop in adult smallpox mortality in London *c*. 1770 was accompanied by a *rise* in smallpox mortality in childhood, and a reduction in the average age of death among child smallpox victims. We argued that this pattern was unlikely to be a consequence of a rise in the popularity of inoculation in either rural or urban populations because inoculation was unlikely to *raise* smallpox mortality. The simplest explanation for these simultaneous changes in child and adult smallpox mortality was that the risk of smallpox infection rose both within London (driving down the average age at infection and raising mortality at younger ages) and in its migrant hinterlands (reducing the number of immigrants who had not encountered smallpox before migration). Therefore the reduction in smallpox as a proportion of burials in London in this period, evident in the burial totals reported in the London Bills of Mortality, was a function of the decline in smallpox among adult migrants, with no accompanying reduction in the importance and lethality of smallpox among the urban‐born population.

Razzell argued in a commentary on our article that our results could be explained solely by the spread of smallpox inoculation within and outside London.^17^ Although he had argued previously that inoculation was ‘greatly neglected in the large towns’,^18^ his commentary provided an extensive survey of anecdotal evidence for inoculation in London which revealed a much more ambivalent picture of the uptake of inoculation in the capital than previously claimed, although the evidence cited *for* the extensive use of inoculation derived mainly from the period after 1790. Razzell also drew on evidence of smallpox burials from the London parish of St Mary Whitechapel to argue, first, that the decline in adult smallpox was not as abrupt as our sources indicated; and, second, that childhood smallpox mortality in fact declined in the last quarter of the eighteenth century. On this basis he concluded that the trends we observed in childhood smallpox rates in St Martin in the Fields were either artifactual or unrepresentative of metropolitan trends.

The gradual decline in adult and child smallpox mortality reported by Razzell was more consistent with the gradual spread of inoculation than were the precipitous changes we reported for St Martin in the Fields and St Dunstan Stepney. Razzell argued that even in London where smallpox was endemic the tendency was to inoculate only when an epidemic threatened. Since smallpox peaked in mortality every two to three years in the second half of the eighteenth century in London periodic surges in inoculation would have left most older children protected and caused smallpox mortality to become concentrated at the youngest ages, producing the shift in age patterns of smallpox mortality reported for St Martin's (but leaving the rise in smallpox mortality at the youngest ages unaccounted for).

While we differ in our interpretations of the patterns observed, it is important to note that there is broad agreement between our and Razzell's observations. All the data presented point to a substantial reduction in the incidence of smallpox among adults in London in the last quarter of the eighteenth century, and among older children. Moreover, we are not in disagreement that the spread of inoculation *outside* London probably played an important role in reducing the susceptibility of adult migrants to London from the 1760s until the adoption of vaccination.^19^

Our main point of disagreement is on the question of whether inoculation of children *in* London contributed to a decline in metropolitan smallpox mortality. Previously we discounted this possibility because we found no evidence for a reduction in smallpox mortality among children before 1800 (although there was a redistribution of risk from older children to infants). Razzell pointed to the weaknesses of the data that we (and implicitly he) have deployed so far, in particular age heaping and rounding and gaps in the series. We reject his claims regarding the importance of defects in our data. While the sources for St Martin's suffer from gaps, the richness of the records allowed us to detect and correct many of the biases that remain unknown in the sources used by Razzell, such as the problems of changes in rates of burial imports and exports.^20^

However, further debate on the relative merits of our sources is unlikely to be decisive. Instead we have collected additional evidence with which to test the hypothesis that inoculation of children in urban areas contributed to a reduction in smallpox mortality before 1800. Razzell argued that ‘[t]he spread of [inoculation] probably occurred gradually in London between 1760 and 1812, which is consistent with the changing age patterns of the disease in Whitechapel and the overall decline of childhood smallpox mortality in the same period’.^21^ Thus he identified two features that he regarded as indicative of the effects of urban inoculation in urban populations: first, the gradualness of the change in the age distribution of smallpox mortality; and second, a reduction in the level of childhood smallpox mortality.

In the rest of this article we use these criteria to assess the likelihood that inoculation made a major contribution to patterns of childhood smallpox mortality in large urban centres before 1800. Section I analyses smallpox mortality in Manchester where we applied the same methodology deployed previously in St Martin in the Fields and obtained similar results (that is, a concentration of mortality at the youngest ages and a rise in the importance of smallpox as a cause of death after *c*. 1770). Section II uses a family reconstitution methodology to obtain more reliable measures of smallpox mortality in the parochial population of St Martin's. This new analysis confirms our earlier findings of a rise in smallpox mortality among young children in the last quarter of the eighteenth century. Section III considers new evidence of a shift in smallpox epidemic frequency in late eighteenth‐century London and considers international evidence for such shifts in other populations. The implications of these new findings are discussed in section IV.

## I

**Figure 1:**
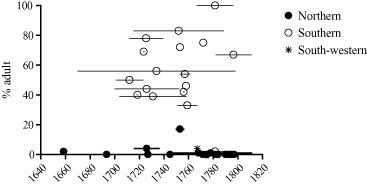
Percentage of smallpox burials aged 10*+* or adult by period and region of Britain *Notes:* Where burial registers did not give age, child status was assigned to burial records described as ‘infant’, ‘son of’, and ‘daughter of’. In rural parishes these descriptors were applied to teenagers resident with a parent as well as younger children. *Sources:* Razzell, *Conquest of smallpox*, pp. xi–xiii.

The geography of smallpox mortality in Britain remains an interesting puzzle. On the basis of his survey of historical smallpox epidemics Razzell argued that smallpox was an endemic childhood disease in the relatively dispersed populations of northern England and mainland Scotland but remained an infrequent epidemic disease affecting all ages in many rural areas of southern England throughout the eighteenth century.^22^ The infrequency of smallpox in the rural south provided a reservoir of susceptible migrants that probably comprised the majority of adult smallpox victims in London. The evidence for an early endemicization of smallpox in northern Britain derives from the rarity of adult smallpox burials in burial records from a number of northern villages and towns. Figure 1 shows the percentage of adult burials for a range of settlements by date and region, collated or collected by Razzell.^23^ Low values indicate that smallpox was primarily a disease of childhood, and that epidemics were frequent. Most of the evidence for southern England dates from the period before 1770, whereas most of the evidence for northern England dates from after 1775, when it became common particularly in Yorkshire for burial registers to include age and cause. Nevertheless, there appears to be a very striking geographical difference in the proportions of adult burials across the entire period 1650–1813. It is very puzzling that smallpox apparently circulated more regularly in northern parts of the country characterized by dispersed settlements and low population densities than in the more densely populated and urbanized south‐east. However, smallpox was also a childhood disease in very lightly urbanized Sweden by the late eighteenth century, suggesting that it was southern England that was exceptional.^24^

A particularly celebrated example of the almost exclusively childhood nature of smallpox mortality in northern England is Manchester, where Percival reported that of 589 smallpox victims in the period 1768–74 only one was aged 10 or over.^25^ This is remarkable given that the adult population of Manchester must have included a high proportion of rural migrants to fuel the rapid growth of the town, and contrasts with the approximately 20 per cent of smallpox burials that were adult in London in this period.^26^ Percival derived his evidence for Manchester from the sextons' burial books of the collegiate church of St Mary, St Denys, and St George and from the Manchester Bills of Mortality.^27^ The collegiate church (now Manchester Cathedral) was the parish church and served not only the township of Manchester but also the very extensive parish of the same name. The parish was *c*. 60 square miles in area and contained a number of townships of which Manchester was the largest. Manchester's population grew rapidly throughout the eighteenth century and accelerated from the 1780s in response to the mechanization of cotton spinning and later weaving. The town functioned primarily as a commercial centre for the cotton industry until the 1790s when mechanization and the availability of steam power favoured the establishment of cotton factories within the township.^28^ The population of the township grew from around 17,000 in 1757 to almost 24,000 by 1774 and to over 70,000 in 1801.^29^

The collegiate church accounted for almost all burials in Manchester town in the early eighteenth century but less than 70 per cent of all burials by 1800 with the tardy establishment of new churches and chapels in response to rapid population growth. The sextons' books recorded age and cause of death for most entries from 1753 to 1791 (although the books do not survive for the full period)^30^ and also for the period 1803–7 (a total of 22,715 burials).^31^ In addition the burial register of the church of St John Deansgate (also in Manchester town) reported age and cause of death for 99.8 per cent of entries in the years 1769–1812.^32^ St John Deansgate opened as a chapel of ease in 1768 and quickly became a major burial ground for the township. The collegiate church sextons' books are marred by gaps but span the second half of the eighteenth century and therefore allow us to test for evidence of a sudden change in the age structure and levels of smallpox burials similar to that which occurred in London. St John Deansgate opened too late to capture mid‐eighteenth‐century conditions but provides an uninterrupted burial series with almost no omission of cause of death or age from 1769 to 1812.

Consistent with the geography of smallpox mortality in figure 1, even in the mid‐eighteenth century 96 per cent of smallpox burials in Manchester were children and only 4 per cent were adult (table 1). This contrasts starkly with the 20 per cent of smallpox burials that were adult in London in this period. This difference in the proportion of adult smallpox burials was not a function of differences in the age structure of the populations of Manchester and London in this period, because adults comprised a similar proportion of all‐cause burials in both populations, and the underlying age structures of the populations at risk were probably similar.^33^ The scarcity of adult smallpox victims in Manchester instead probably reflects its much smaller migration field. Creighton considered London unusual compared with other English towns in receiving ‘a constant recruit direct from the country … from parishes where, as Lettsom says, “the smallpox seldom appears” ’, a pattern which he thought explained the atypical preponderance of adult smallpox victims in London in the eighteenth century.^34^ Notwithstanding Manchester's limited migration field, the paucity of adult victims implies high levels of smallpox infection not only within Manchester but also in the rural areas which supplied migrants to the town.

**Table 1:** Percentage age distribution of smallpox burials in Manchester

Age (completed years)	St Mary, St Denys, and St George collegiate church				St John Deansgate	
	1753–61	1772–8	1785–91	1803–7	1769–99	1800–12
0	18.9	32.7	29.2	32.3	23.4	22.9
1–4	69.9	62.1	66.9	63.7	73.1	73.7
5–9	7.5	3.9	2.8	2.6	2.7	1.9
10–19	1.5	0.8	0.7	0.2	0.2	0.4
20+	2.2	0.4	0.4	1.1	0.4	1.1
Cause not given	5.4	6.1	2.7	28.0	0.2	0.0
Adult (10+)	*3.8*	*1.2*	*1.1*	*1.3*	*0.6*	*1.5*
N	734	716	1,731	430	1,261	262

*Notes:* Burials of unknown age were distributed to age groups according to the age distribution of burials of known age. Burials with no cause of death information were distributed to smallpox and other causes according to the distributions of burials with given cause. N refers to the no. of smallpox burials explicitly so described in the source.

Apart from this striking difference in the proportions of adult smallpox burials, the chronology of changes in the age patterns of smallpox burials in Manchester was remarkably similar to those we observed in St Martin in the Fields. As in London, there was a drop in the proportion of adult burials sometime between 1762 and 1771, and only 0.6–1.5 per cent of smallpox burials were aged 10 and over in Manchester after 1771 (table 1).^35^ This proportion was similar in the periods 1772–78, 1785–91, and 1803–7, suggesting that the change in age distribution occurred fairly abruptly in the gap 1763–71. Adults comprised a similarly low proportion of smallpox burials at St John Deansgate in the period 1769–1812 (table 1). As in London, the drop in the proportion of adult smallpox burials was accompanied by a change in the sex ratio of adult smallpox burials. In St Martin in the Fields smallpox burials were male‐biased before 1770, but assumed a normal sex ratio in the last quarter of the eighteenth century.^36^ This male bias in the earlier period was consistent with the limited evidence that male migrants migrated further on average than female migrants and hence that a higher proportion of male migrants to London may have derived from more remote areas than was the case for female migrants.^37^ In Manchester a similar male bias was observed before the 1770s, with males accounting for 75–83 per cent of adult smallpox burials in the period 1753–62.^38^ After 1771 adult smallpox burials were distributed almost equally between males and females. One cause of the high sex ratio in the first period was the large number of soldiers recorded as dying of smallpox in Manchester (12 of 32 adult burials).^39^ This finding is consistent with Landers's hypothesis that smallpox epidemics were often associated with demobilization, although the dates of the soldiers' burials did not coincide with periods of major demobilization.^40^ Rather the apparent over‐representation of soldiers among adult smallpox victims probably reflects the wider geographical origins of troops stationed around Manchester compared with other migrants to the town. After 1771 only two adult smallpox burials were described as soldiers.

**Figure 2:**
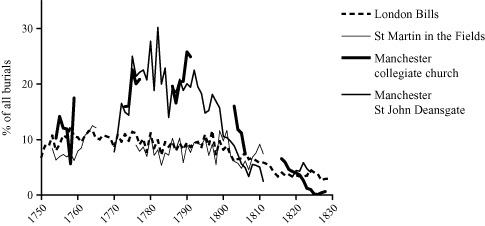
Percentage of all burials described as smallpox, three‐year moving means *Sources:* As for tab. 1; Marshall, *Mortality*, unpaginated tabs.

Smallpox was apparently a much more significant cause of death in Manchester than in London, accounting for up to 40 per cent of all burials in some years. Figure 2 plots three‐year moving means of the proportion of all burials attributed to smallpox in Manchester and in London. Smallpox accounted for over 20 per cent of burials in Manchester in the last quarter of the eighteenth century, almost double the percentage in London. The short run of data from the collegiate church for the years 1753–61 suggests that smallpox may have been a less significant cause of death at mid‐century, accounting for closer to 10 per cent of burials in this period, a level comparable with London. Significantly, smallpox only fell decisively as a proportion of burials in both cities after *c.* 1800, coincident with the advent of vaccination.

Smallpox was apparently endemic in both London and Manchester (that is, maintained by transmission within the population without requiring re‐introduction from outside) and smallpox burials occurred in most weeks. Despite Manchester's much smaller population (less than 17,000 in 1750 compared with *c.* 675,000 in London),^41^ the frequency of smallpox epidemics was similar (roughly biennial) in both cities in the last quarter of the eighteenth century.^42^ To investigate the consequences of these patterns for the risk of smallpox among long‐term urban residents, we must focus on children, most of whom would have been urban‐born. Table 2 shows the age distribution of smallpox burials aged under 10 years in Manchester and in the London parishes of St Martin in the Fields and St Dunstan Stepney for several periods in 1752–99. Surprisingly, even in the mid‐eighteenth century the age distribution of child smallpox victims was much younger in Manchester than in London. However, there was a further concentration of smallpox mortality at the youngest ages in the last quarter of the century (table 2), and the shift seems to have occurred in the period between 1763 and 1772, coincident with the fall in adult smallpox burials in Manchester and in London. The fall in the average age at death from smallpox after 1770 was more pronounced in Manchester, and less than 5 per cent of child burials were aged five and over in the period 1775–99 compared with 10 per cent or more in London.

**Table 2:** Percentage age distribution of child smallpox burials (aged under 10 years)

Age (completed years)	Collegiate church, Manchester			St John Deansgate, Manchester	St Martin in the Fields, London		St Dunstan Stepney, London
	1753–61	1772–8	1785–91	1769–99	1752–66	1775–99	1775–99
0	19.6	33.1	29.5	23.6	17.3	24.7	24.4
1	32.5	30.3	33.7	36.5	21.8	25.6	20.6
2	19.0	18.3	20.4	20.9	18.0	18.5	19.0
3	11.7	10.8	10.0	11.8	17.0	12.9	14.4
4	9.4	3.5	3.5	4.6	12.1	8.3	9.3
5–9	7.8	3.9	2.8	2.7	13.8	10.0	12.3
N	707	659	1,712	1,251	857	1,904	937

*Notes:* Burials of unknown age were distributed to age groups according to the age distribution of burials of known age. Burials with no cause of death information were distributed to smallpox and other causes according to the distributions of burials with given cause. N refers to the no. of smallpox burials explicitly so described in the source.

Smallpox was a more important cause of death in Manchester compared with London in the late eighteenth century (figure 2), and was more concentrated in early childhood (table 2). Taken together, these patterns suggest that smallpox mortality in childhood may have been higher in the smaller town. Alternatively, smallpox may have been a more important cause of death in Manchester because there were fewer deaths from other causes in Manchester compared with London. We do not have census‐type data giving the age structure of Manchester or St Martin in the Fields before 1841 and therefore cannot calculate age‐specific mortality rates to compare between the two populations, except in the case of infants where baptisms can be used as the denominator for the infant mortality rate. However, the problems of calculating infant mortality rates using infant burials and baptisms are well‐known and are particularly acute in the case of Manchester. The collegiate church was the parish church and claimed a right to all fees for marriage, baptism, and burial in the parish. Registration of events at other churches was therefore supposed to incur a double fee that included a fee to the collegiate church.^43^ For this reason it appears that even as Manchester grew approximately seven‐fold in population between *c.* 1750 and 1820, a very high proportion of all marriages and to a lesser extent baptisms continued to be registered at the collegiate church. Burials, however, presented a greater logistical problem for the collegiate church with the progressive over‐crowding of burial grounds, and its monopoly on burials was eroded over the eighteenth and especially nineteenth centuries. Therefore the ratio of burials to baptisms at the collegiate church declined over the eighteenth century, and baptisms at the church came to represent a much larger proportion of the Manchester population than burials. In this case infant mortality rates would appear to decline progressively simply as a consequence of an expansion in the population at risk of baptising compared with burying at the collegiate church. Notwithstanding this increasing bias over time, it is instructive to note that although infant mortality calculated in this way was unrealistically low, smallpox‐specific infant mortality rates derived for Manchester were still higher than rates in St Martin in the Fields (which doubled from 13 to 24 burials per 1,000 baptisms between the third and fourth quarters of the eighteenth century) (table 3).^44^

**Table 3:** All‐cause and smallpox infant mortality rates at the collegiate church, Manchester

Period	Total infant burials	Infant smallpox burials	Baptisms	Infant mortality rate (burials per 1,000 baptisms)	Smallpox infant mortality rate (per 1,000 baptisms)
1753–61	932	146	5,872	159	25
1772–8	1,067	236	7,037	152	34
1785–91	2,431	518	14,452	168	36
1803–7	1,457	197	16,811	87	12

*Notes:* Infant burials were adjusted for burials of unknown age and infant smallpox burials were adjusted for burials of unknown age and cause as for tab. 1.

The progressive divergence of burial and baptism practices in Manchester makes it impossible to test whether infant smallpox mortality rose in Manchester as in St Martin in the Fields after 1770. Table 3 suggests that smallpox mortality rose in infancy even as total measured infant mortality fell as the collegiate church buried a diminishing fraction of the township's population. We can however test whether smallpox increased its relative share of deaths. A striking feature of table 4 is the increase in smallpox as a proportion of all causes of death in childhood in the period after *c.* 1770. In Manchester, smallpox accounted for nearly a quarter of all burials under age 10 before 1770, but almost a third of all burials in this age group in the period 1772–91. At the ages where smallpox mortality was concentrated, the age group of one to four years, smallpox accounted for around a third of all burials in the mid‐eighteenth century but for over 40 per cent of all burials at these ages between 1772 and 1791. This proportion fell again after 1800 (halving in St John Deansgate), probably as a consequence of vaccination. While in London smallpox was given as the cause of death in 4 per cent of infant burials in St Martin in the Fields in the mid‐eighteenth century, it accounted for 16 per cent in Manchester in the same period, and nearly 30 per cent of deaths at age one.^45^ As in St Martin in the Fields and the London Bills, smallpox only fell decisively as a proportion of all burials after 1800 (table 4 and figure 2).

**Table 4:** Smallpox burials as a percentage of all burials

Age (completed years)	Collegiate church, Manchester				St John Deansgate, Manchester		St Martin in the Fields, London		
	1753–61	1772–8	1785–91	1803–7	1769–99	1800–12	1752–66	1775–99	1802–12
0	15.7	22.1	21.3	13.5	19.8	7.9	4.0	6.8	6.7
1	27.5	40.4	45.5	27.1	43.1	22.4	17.9	17.9	10.6
2	30.7	42.7	47.8	30.6	50.2	22.2	27.2	26.0	17.9
3	30.5	45.9	53.0	29.7	44.5	22.2	41.7	32.2	24.8
4	40.0	30.8	44.1	26.0	33.7	16.9	41.3	32.4	25.1
5–9	15.2	20.6	18.1	10.6	12.1	2.8	33.5	22.8	13.2
<10	23.9	31.1	33.6	20.2	32.5	14.1	13.6	14.5	11.4
No cause	5.4	6.1	2.7	28.0	0.0	0.0	0.8	7.7	6.3

*Notes:* Infant burials were adjusted for burials of unknown age and infant smallpox burials were adjusted for burials of unknown age and cause as for tab. 1.

The rise of smallpox as a cause of death in childhood in the late eighteenth century is difficult to reconcile with any significant beneficial effect of inoculation. Nonetheless it is possible that such a rise was not a function of a rise in the level of smallpox infection or mortality but rather reflected a larger reduction in other causes of death. We think this is unlikely, because the change was fairly abrupt (confined to the period between 1763 and 1772), and because there was no corresponding fall in all‐cause infant mortality that would suggest improvements in mortality from causes other than smallpox or changes in the recording of burials by age (table 3). It is possible that the very rapid growth of Manchester, accompanied as it was by changes in the density and composition of the population, favoured an increase in smallpox as a share of child deaths. However, we think this is unlikely given that the major shift in the age structure and importance of smallpox mortality appears to have occurred *c*. 1770, before the most rapid phase of Manchester's growth and physical expansion.

In conclusion, the evidence from Manchester provides no support for the argument that urban child smallpox mortality was reduced before 1800 as a consequence of inoculation. Manchester seems to have experienced changes in smallpox mortality very similar to those reported for St Martin in the Fields and St Dunstan Stepney: a fall in the proportion of adult smallpox burials after *c*. 1770, a shift in the age distribution of child smallpox burials to younger ages, and a rise in the relative importance of smallpox as a cause of death in early childhood. These changes seem to have occurred fairly abruptly, probably sometime in the 1760s. Percival commented in 1773 with reference to Manchester that ‘inoculation is not much practised here’ and his subsequent efforts to promote mass inoculation were widely considered a failure.^46^ Instead smallpox infection appears to have intensified in Manchester in the late eighteenth century and to have become a more significant cause of death in early childhood.

## II

The evidence presented for Manchester in section I suffers from the same potential biases as that presented previously for St Martin in the Fields, St Dunstan Stepney, and St Mary Whitechapel. In each case we lacked a precise denominator for the mortality rates, and although in the case of Manchester and St Martin's we used all‐cause burials by age as an indicator of changes in the underlying population, it remained possible that unobserved changes in the age structure or size of the population were driving apparent changes in mortality rates. Here we present data that overcome this problem by the use of strict rules for determining the size and composition of the population at risk (a technique known as ‘family reconstitution’).^47^ The burial day books and christening fee books survive for St Martin in the Fields for the period 1752–1812 and have been used to create a partial family reconstitution of the parish. The reconstitution is ‘partial’ for two reasons. First, because few couples remained in the parish from marriage until death, almost all families in the reconstitution are incomplete (that is, not all births are included). Second, the size of the parish population (*c*. 27,400 in 1801) and high levels of residential mobility made it necessary to confine the calculation of mortality to young children, where age at burial was given with remarkable precision and where the date of birth (and in some cases address) permitted greater confidence in the accuracy of linkages between burial and baptism records.

The high quality of the records for St Martin in the Fields also revealed the extent of a number of well‐rehearsed problems associated with family reconstitution methodologies applied to urban populations.^48^ The most serious problems were, first, the tendency for families to move in and out of observation through small‐scale moves across parish boundaries, and second, for infants to be sent out of the parish, either dead or alive. To investigate the first problem we used street address at baptism to determine the frequency of small‐scale moves within the parish: the frequency was sufficiently high that it proved necessary to restrict analysis to observations of families in residence at a single street address, and to exclude families from analysis at the point where there was evidence of a residential move. When analysis was restricted to apparently ‘stable’ families, then capture of birth events seemed relatively complete (as judged by birth intervals and proportions of births that were multiple births). However, even under these conditions it was evident that some infants baptized in the parish escaped observation sometime after baptism although their families remained in observation and continued to baptize infants in the parish. This second problem probably arose either because infants were sent away from their families to be nursed in other parishes (often rural), or because corpses were exported for burial in other parishes. The sextons' books documented burials of St Martin's parishioners in other parishes and these recorded exported burials were included in the family reconstitution. However, searches of burial registers of adjacent parishes revealed a number of burials of St Martin's residents that were not recorded in the St Martin's sextons' books. Therefore it is likely that some proportion of exported burials went unobserved and escaped inclusion in the family reconstitution, producing an underestimate of mortality rates. The escape of infants from observation, either to nurse or at burial, could be estimated using birth interval analysis and these estimates used to adjust observed mortality rates. The methodology used is detailed elsewhere.^49^

Table 5 presents the risk of dying from smallpox and from other causes for young children of reconstituted families in St Martin's.^50^ We present rates for the first two years of life, ages for which linkage of burials to baptisms was most secure. Four sets of rates are presented. The first is the smallpox mortality rate for each period of good cause of death recording, unadjusted for omission of cause of death or missing infants. The second set of rates is adjusted only for omissions of cause of death information. The third and fourth sets of rates were further adjusted to correct for ‘missing’ infants. The third set consists of mortality rates adjusted on the assumption that most missing infants were sent out of the parish to be wet‐ or dry‐nursed elsewhere. We reduced the size of the population at risk by the number of infants estimated to be missing, without making any further assumptions about the mortality rates of infants nursed extra‐parochially. The alternative assumption, that missing infants died and were exported from the parish without record in the sextons' books, was modelled by adding the deaths of these missing infants back into the life tables used to calculate the fourth set of rates. The latter rates of mortality are improbably high. However, the actual mortality rates among children of the reconstitution families were probably a function of both extra‐parochial nursing and some low level of unobserved burial export as well as observed mortality. Importantly, trends over time were similar regardless of the type of adjustment.

**Table 5:** Smallpox and all‐cause mortality rates in St Martin in the Fields derived from family reconstitution

Age (months)	Probability of dying in age interval *1,000								
	Smallpox			All‐cause			Smallpox % of all‐cause		
	1752–66	1775–99	1800–12	1752–66	1775–99	1800–12	1752–66	1775–99	1800–12
Unadjusted									
0–5	4.0	6.5	3.7	179.7	130.4	99.8	2.2	5.0	3.7
6–11	9.5	13.7	7.6	72.2	69.9	55.7	13.2	19.6	13.7
12–23	13.6	23.4	5.8	105.4	106.5	75.8	12.9	22.0	7.6
0–23	26.9	43.0	17.0	319.1	277.3	214.3	8.4	15.5	7.9
Adjusted for missing causes of death									
0–5	4.9	7.2	3.8				2.7	5.5	3.8
6–11	13.3	15.4	7.8				18.5	22.0	14.0
12–23	18.1	26.8	6.1				17.2	25.2	8.0
0–23	35.1	48.6	17.6				11.0	17.5	8.2
Adjusted for missing causes of death and missing infants (presumed sent to nurse)									
0–5	5.5	7.8	4.1	200.1	141.7	106.6	2.7	5.5	3.8
6–11	16.2	17.8	8.9	87.7	80.7	63.4	18.5	22.0	14.0
12–23	23.7	31.2	6.9	137.7	124.0	86.7	17.2	25.2	8.0
0–23	40.8	54.1	19.4	370.7	308.8	235.7	11.0	17.5	8.2
Adjusted for missing causes of death and missing infants (presumed exported for burial)									
0–5	9.6	13.5	7.6	349.2	246.2	199.6	2.7	5.5	3.8
6–11	26.8	27.9	14.8	145.1	126.6	105.5	18.5	22.0	14.0
12–23	31.4	43.9	11.5	182.4	174.2	144.1	17.2	25.2	8.0
0–23	59.9	79.9	31.9	545.1	456.3	387.2	11.0	17.5	8.2

*Notes:* All‐cause rates were adjusted for the unobserved effect of infants removed from observation *alive* by subtracting the missing infants (as calculated from birth interval analysis) from the population at risk at ages 15 and 182 days in the proportions 10% and 90% of missing infants. This was a crude adjustment based on the average age at baptism and average length of birth intervals: changes in the ages at which infants were subtracted did not affect calculated rates markedly. All‐cause rates were adjusted for the unobserved effect of infants removed from observation *dead* by adding missing infant burials to deaths at ages 15 and 182 days in the proportions 10% and 90% of missing infants. Smallpox rates were adjusted by multiplying the rate adjusted only for missing causes by the ratio of the adjusted to unadjusted all‐cause rates. The ratio of smallpox mortality to all‐cause mortality was the same for all adjusted rates because the method used to adjust smallpox rates for missing infants was to assume that the effect of missing infants on the smallpox mortality rate was the same as for all‐cause mortality.

Regardless of the method used to adjust mortality rates, there was a rise in smallpox mortality rates in all age groups under two, and the probability of dying of smallpox in this age range rose by over 30 per cent between the third and fourth quarters of the eighteenth century (table 5, adjusted rates, cols. 2–3). This rise in mortality was apparently specific to smallpox because the overall probability of dying fell in the first six months of life and was fairly stable at ages 6–23 months (table 5, cols. 5–6). Therefore the share of mortality attributable to smallpox rose at ages 0–1 in the period 1775–99 (table 5, cols. 8–9). The fall in all‐cause infant mortality was driven mainly by a fall in mortality in the first month of life that was associated with a lengthening of birth intervals that suggested that maternal breastfeeding became more common. Any increase in the prevalence of maternal breastfeeding is likely to have had positive effects on infant and child health throughout the first two years of life. Therefore the rise in smallpox mortality after *c.* 1770 cannot be attributed to any general worsening of health. The specific rise in smallpox relative to other causes of death also suggests that this result is not an artefact of measurement biases or of changes in the composition of the sample.^51^

Critically, smallpox mortality among young children in the reconstitution sample only fell after 1800, when vaccination would have become available (table 5). These reductions coincided with the rapid declines in smallpox burials in the parish of St Martin in the Fields, in the London Bills, and in Manchester (figure 2).

The apparent rise in smallpox mortality among young children in St Martin in the Fields contrasts with evidence from Landers's study of London Quakers where smallpox mortality fell among children of all ages in the second half of the eighteenth century.^52^ We suggested previously that Quakers may have been precocious in the adoption of inoculation. Among Landers's Quaker sample, smallpox burials fell as a proportion of child burials after 1750, indicating a genuine and specific fall in smallpox mortality. In the case of St Martin's, smallpox mortality fell only after *c*. 1800, and then it fell both absolutely and as a proportion of all mortality. The most parsimonious explanation of these trends is that smallpox mortality in young children only fell decisively with vaccination in London, although some social groups (including London Quakers) probably adopted inoculation before 1800.

## III

Further evidence for a fairly abrupt change in the dynamics of smallpox mortality around 1770 comes from recent mathematical modelling of smallpox burials in London. In common with a number of other viral diseases, historical smallpox mortality does not conform to simple mathematical models of immunizing epidemic diseases. In the simplest case, the frequency of epidemics in a population depends on the size of the population and biological and social factors affecting the transmission rate of the pathogen.^53^ Once an immunizing disease becomes endemic within the population (that is, constantly circulating without requiring reintroduction) then oscillations in mortality should be dampened (that is, epidemic cycles should become progressively smaller in amplitude). By the mid‐eighteenth century smallpox was clearly endemic in London, with deaths from smallpox reported in most weeks. However, superimposed on this pattern was an epidemic cycle of peaks and troughs in smallpox burials that did not dampen over time but grew progressively shorter in wavelength. This type of pattern is usually considered to indicate some factor ‘forcing’ epidemics even in endemic conditions, for example, annual patterns of school terms in the case of measles, seasonal migration patterns, or periodic changes in food availability affecting susceptibility.^54^ In our original article we were puzzled that the significant changes we identified in the age patterns of smallpox mortality in London *c*. 1770 did not coincide with any obvious change in the periodicity of smallpox epidemics, and we speculated that this forcing mechanism was insensitive to the changes we proposed. Our assessment was based on the spectral analyses of *annual* smallpox burial counts conducted by Duncan, Duncan, and Scott.^55^ However, recent wavelet transform analysis of *weekly* smallpox burial totals by Krylova provides a much higher‐resolution picture of epidemic cycles.^56^ Krylova concluded that there was a shortening of epidemic cycles *c* .1768. This change in frequency from a dominant three‐year cycle to a two‐ to three‐year cycle was accompanied by the emergence of a strong annual cycle and a shift in the seasonality of epidemics from winter to autumn and winter.^57^

Krylova attributed these changes to inoculation because of the coincidence in timing between the introduction of Suttonian inoculation and the shifts in smallpox epidemic cycles. However, she did not specify how an essentially preventative practice might produce these effects. Instead she noted that inoculation did not reduce transmission rates or the number of susceptible individuals.^58^ In contrast the introduction of vaccination resulted in a progressive and dramatic decrease in the frequency of epidemics from *c*. 1808 consistent with a strong reduction in the number of susceptible individuals. Therefore to the extent that inoculation could have caused the observed changes in smallpox dynamics after *c*. 1768, its effects on the metropolitan population must have been very different from those of vaccination. If inoculation had reduced smallpox mortality by increasing the degree of artificial immunity in the population, then the reduction in susceptible individuals would be expected to have reduced epidemic frequency (as was the case with vaccination). It is possible that any beneficial effects of inoculation were masked with respect to smallpox epidemic frequency by increases in the rate of population growth in London, driven by both increased immigration and improving survival rates in the last quarter of the eighteenth century. However, these processes probably accelerated after 1800 but did not prevent a significant impact of vaccination on epidemic frequency. In any case Krylova's analysis provides little evidence that inoculation operated to reduce smallpox mortality or susceptibility in late eighteenth‐century London.

The timing of these changes in the frequency and seasonality of smallpox epidemics in the London Bills of Mortality *c*. 1768 is consistent with a sudden increase in the rate of transmission of smallpox. Such a change could have been caused by a rise in the infectiousness (or R_0_ value) of the virus or by enhanced dissemination of infection via inoculated individuals. The latter is unlikely given the suddenness of the change in epidemic frequency detected by Krylova, and we think her analysis supports a fairly abrupt change in the transmission properties of the smallpox virus. Unfortunately there are few data with which to test this hypothesis.

**Figure 3:**
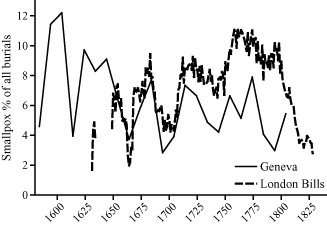
Smallpox burials as percentage of all burials, Geneva and London Bills of Mortality *Notes:* % are decennial averages for Geneva and five‐year moving means for London. *Sources:* Perrenoud, *La population du Genève*, p. 462; Creighton, *History*, pp. 436–7, 456, 461, 531, 535, 568.

Although the smallpox virus is considered to be very stable genetically, there is rather speculative historical evidence for several historical shifts in the properties of circulating strains. An increase in virulence in England in the seventeenth century is suggested by the rise in the frequency and death toll of smallpox epidemics in the London Bills and by accounts of contemporary medical writers.^59^ Perrenoud drew attention to what he regarded as the strong coincidence in patterns of smallpox mortality between London and Geneva before vaccination, a synchrony which he attributed to the agency of related non‐anthropogenic factors in both cities (figure 3).^60^ He also demonstrated a rise in smallpox mortality in the first year of life in the last quarter of the eighteenth century in Geneva. The appearance among the victims for the first time of significant numbers of neonates induced Perrenoud to argue that a novel strain of smallpox may have emerged *c*. 1777.^61^ He also noted a sudden shift in the seasonality of smallpox mortality in the last quarter of the eighteenth century, from a less marked seasonal pattern to an autumn peak, consistent with the seasonal shift detected by Krylova for London.^62^

Other evidence for changes in patterns of smallpox mortality in the second half of the eighteenth century is more equivocal. As described previously, case‐fatality rates increased markedly in the London Smallpox Hospital and among the uninoculated in Boston, US, in the last quarter of the eighteenth century.^63^ However, while a rise in virulence could be associated with a rise in infectiousness if both depended on the amount of virus produced in the host, there is no strict connection between infectiousness and virulence and it is very possible that an increase in transmission rates occurred that was accompanied by no change or even a decline in virulence and so went unremarked by most observers.^64^

**Figure 4:**
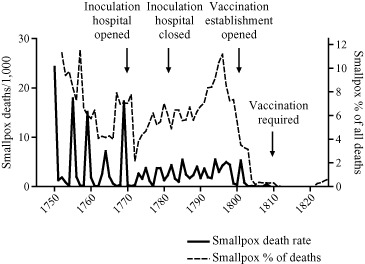
Annual smallpox mortality and percentage of all deaths, Copenhagen *Source:* Vaccination Commission, *First Report* (P.P. 1889, XXXIX), app., pp. 107–8.

Smallpox mortality appears to have declined substantially in Sweden and Denmark in the last quarter of the eighteenth century, but the relatively scant evidence for widespread inoculation has led some authors to attribute these declines to autonomous factors such as viral shift or climatic variations.^65^ In Copenhagen the opening of a new inoculation clinic coincided with a sudden shift in the pattern of smallpox epidemics, from very large epidemics at roughly five‐yearly intervals to a more muted pattern of small roughly biennial epidemics superimposed on a higher background of smallpox mortality (figure 4). The sudden change from an epidemic to an endemic pattern was associated with a virtual halving of crude smallpox mortality from 4.8/1,000 in the period 1750–69 to 2.6/1,000 in 1770–1801.^66^ Despite this compelling coincidence it remains disputed whether inoculation was responsible for this sudden change, because the clinic itself was closed in 1783, for reasons unknown, and Johansen has argued that inoculation was very limited.^67^ Moreover, the speed of the transition and the subsequent stability of the new pattern would imply a sudden mass adoption of inoculation in Copenhagen in contrast to its more gradual adoption elsewhere. The shift to a more endemic pattern could have occurred as a result of increased smallpox circulation associated with inoculation, but could also reflect a change in the transmission properties of the virus. As in the case of London, the decisive reduction in smallpox mortality coincided with the introduction of vaccination in the first decade of the nineteenth century.

**Figure 5:**
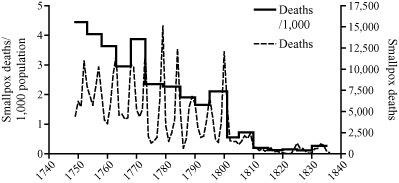
Annual smallpox deaths and quinquennial smallpox mortality, Sweden *Source:* Sköld, *Two faces*, pp. 52, 541–57.

Sweden is the only country for which we have cause of death data for the national population in the eighteenth century. Smallpox mortality apparently halved between 1750 and 1800 (figure 5), despite relatively thin evidence for any impact of inoculation.^68^ The proportion of deaths attributed to smallpox fell from 13–14 per cent in 1754–63 to 8.3 per cent by 1796–1801 (the equivalent figures for Stockholm are 8 and 5.4 per cent), indicating a specific decline in smallpox mortality. However, before 1773 measles deaths were included in smallpox statistics and this must be considered in judging the extent of mortality decline. Sköld and Fridlizius estimated that measles accounted for no more than 10–15 per cent of deaths due to both causes.^69^ However, examination of the annual deaths due to smallpox indicates a sudden reduction by around two‐thirds in the baseline mortality from smallpox in non‐epidemic years after 1773 (figure 5). The sudden change in 1774 suggests that measles may have accounted for much of the higher mortality attributed to smallpox before 1774, or that some other change in smallpox mortality coincided with the change in registration practice. In Finland, a part of Sweden and under the Swedish registration system until 1809, there was a rise in smallpox mortality and in the frequency of epidemics in the last quarter of the eighteenth century, and smallpox rose from 8 per cent of all deaths in 1751–75 to 11 per cent of all deaths in 1776–1800.^70^

Although the variola virus has a very low mutation rate, its origin in humans appears to be sufficiently ancient for several major variants to have arisen. Data collected during the global smallpox eradication programme in 1959–80 revealed a number of distinct smallpox strains with case‐fatality rates ranging from 0.2–1 per cent in the case of the mild variola minor strain to 20–30 per cent in the case of Asian variants of variola major.^71^ Phylogenetic analysis of strains circulating in the mid‐twentieth century suggests that a number of different smallpox variants existed in endemic form in different parts of Eurasia and Africa before the sixteenth century. These different strains presumably evolved from an ancestral strain through processes of isolation and genetic mutation, but at least from the sixteenth century migration, trade, and conquest resulted in the spread of multiple strains of smallpox both to the New World and between regions of the Old World.^72^ In the mid‐twentieth century variola minor was still endemic in some parts of Europe that experienced only occasional introductions of epidemic variola major, and variola minor and major strains co‐existed in some African populations.^73^ The co‐circulation of multiple strains could provide one explanation for the large variations in virulence between epidemics noted by many medical writers in seventeenth‐ and eighteenth‐century England.^74^ Unfortunately for historians, endemic smallpox was eliminated from Europe too early for the genetic strains involved in pre‐twentieth‐century epidemics to be identified. Here it is sufficient to note that changes in the properties of circulating smallpox viruses could have arisen via mutation or recombination of an existing strain or via importation of a novel variant, and that the sudden introduction of new strains has occurred before in the history of smallpox.

## IV

The extent to which smallpox inoculation was practised in urban areas has important implications for our understanding of the transformation of urban life expectancies in the late eighteenth century. Smallpox accounted for 10–20 per cent of burials in British cities in the second half of the eighteenth century, and although vaccination profoundly reduced smallpox mortality in the nineteenth century the mass uptake of vaccination was generally too late to explain the reversal of the ‘urban graveyard’ phenomenon which was evident in England from the 1770s.

Here we have reported analyses of a range of quantitative sources from both London and Manchester using several techniques (event history analyses as well as simpler calculations of rates and proportions from aggregate data for Manchester) that confirm the validity of our original findings, first, that there was a shift in the age structure of urban smallpox burials around 1770; second, that this shift was abrupt and affected urban‐born children as well as adult migrants; and third, that smallpox mortality rose in younger children as the average age at smallpox burial fell.

In both London (St Martin in the Fields and St Dunstan Stepney) and Manchester there was an abrupt fall in the number and proportion of adult smallpox burials after *c*. 1770. Simultaneously smallpox mortality rose in young children in St Martin in the Fields. Smallpox rose in importance as a cause of death among young children in Manchester in the same period. This rise was probably accompanied by a rise in smallpox mortality rates among infants in Manchester, but the excessive registration of baptisms relative to burials at the collegiate church made this difficult to establish. These data do not support Razzell's claims regarding the importance of smallpox inoculation to mortality decline in urban areas in the late eighteenth century.

We do not dispute the claim that smallpox inoculation contributed to a reduction in the susceptibility of migrants to urban areas. Most urban‐born adults would have been survivors of smallpox in childhood and by the mid‐eighteenth century adult victims of smallpox in London and Manchester were predominantly migrants from relatively remote rural areas. Inoculation against smallpox probably made a significant contribution to the reduction in smallpox mortality of these migrants in the last quarter of the eighteenth century, and the reduction of smallpox in this distinct sub‐population would have reduced smallpox burial totals in urban areas. Whether these adults were inoculated before migration or after arrival in urban areas is a moot point that we cannot address with the data we have. However, our analyses do not support the argument that inoculation reduced smallpox mortality among children in urban areas. There was a clear shift in the age structure of smallpox burials to younger ages after 1770 to the advantage of older children. However, reductions in smallpox mortality in later childhood were accompanied by a worsening of smallpox mortality in early childhood in St Martin in the Fields and probably also in Manchester.

Was smallpox inoculation widely practised in urban areas? Contemporary accounts of inoculation in urban centres in the eighteenth century provide conflicting accounts of the popularity or otherwise of the practice. While we could not detect any evidence of a benefit of inoculation to urban‐born children it remains possible that inoculation was prevalent but was ineffective or actually increased smallpox mortality. In theory inoculation could increase the circulation of smallpox by increasing the number of infected individuals. This could increase the frequency of smallpox epidemics (as detected by recent analyses of weekly metropolitan smallpox burials) and so lower the average age at infection and raise smallpox mortality at younger ages. Such a scenario is a theoretical possibility and was used by a number of high‐profile opponents of urban inoculation. However, such an explanation seems inconsistent with the suddenness of the shift in age structure, which occurred within a 10‐year period in both London and Manchester, and its subsequent stability until *c*. 1800.

In our original article we proposed an alternative explanation for the suddenness of the shifts in age structure and the rise in smallpox mortality in infancy after 1770. We suggested that there may have been a shift in the properties of the viral strain circulating in the English population that increased the rate of transmission of smallpox. Increased infectiousness would have favoured rapid spread of the novel strain and could have produced the sudden change in the age structure of burials and the frequency of epidemics. An increase in the probability of infection should have driven down the average age at infection, raising the risks of infection and death at younger ages even as older children and adults enjoyed a greater freedom from the disease as survivors of earlier attacks. On balance we think the weight of new evidence supports this scenario, however intellectually unappealing the resort to exogenous change as an explanatory factor.

The dynamics of historical smallpox transmission remain a puzzle. Analysis of a range of high frequency burial series and age‐specific rates for a variety of settlement types is necessary to tease out the factors driving the curious geographical patterns of smallpox burials evident in figure 1. Figure 1 suggests that the curious divide in smallpox patterns between a childhood disease in northern Britain and an infrequent epidemic disease in southern communities persisted throughout the eighteenth century. Recently Razzell provided further examples of the persistence of adult susceptibility in southern villages into the last decades of the eighteenth century, as evidence against the possibility of a rise in smallpox infectiousness.^75^ However, the persistence of susceptible adults provides as little support for the importance of inoculation as it does for an argument of increased infectiousness. If at least half the population of Brighton required inoculation in 1786 and at least 37 per cent in 1794, then clearly inoculation had not penetrated widely into the rural populations from which Brighton drew its immigrants. It is likely that neither inoculation nor a process of viral change was sufficient to expose the entire population to smallpox, natural or acquired. Despite the sudden decline in adult smallpox burials in London, 5 to 10 per cent of smallpox burials were adult even in the last quarter of the eighteenth century. This is much higher than the 1 per cent of smallpox burials in Manchester in the same period and indicates that the rural reservoirs of migrants to London remained incompletely integrated into a national disease environment in this period.

Finally, the conclusion that inoculation had little demographic impact on the urban‐born before *c.* 1800 returns us to the question of why inoculation was not practised more widely in urban populations. We have found no evidence at all that inoculation was carried out by the parish of St Martin in the Fields in the eighteenth century and only one reference to a payment made for inoculating children—at nurse at Teddington in 1804.^76^ The parish seems, in striking contrast, to have adopted vaccination with alacrity. Wholesale vaccination of workhouse children was ordered in 1813 and in 1815 and 1817 parents who refused to have children vaccinated were to be expelled along with their offspring.^77^

In the case of St Martin in the Fields the costs to the parish of naturally acquired smallpox seem to have been small. The parish workhouse, erected in 1725, functioned partly as a hospital for the parish poor and although it appears that smallpox sufferers were usually excluded, a small number of (usually adult) inmates were noted as infected with smallpox at admission, and smallpox was an occasional cause of death among long‐term child inmates. A ward for smallpox sufferers was constructed in 1736, but this was probably intended only for adults.^78^ Since urban‐born victims were mainly children who would have been nursed at home, the costs of the disease to the parish were probably minor. Poor families afflicted with smallpox would normally have been relieved outdoors, but it seems unlikely that this would have been a significant cost in most years. In 1726/7 when the overseers' accounts are relatively detailed, out of 4,731 payments made to the outdoor poor, just 19 referred explicitly to smallpox sufferers. The disease was just one of a range of illnesses mentioned by overseers and by no means the most common: generic sickness was reported among the poor in 584 entries in that same year.^79^ Smallpox cannot have been a priority, either for the parish or for parents whose children faced a great variety of threats.

Burying smallpox victims, as opposed to relieving them, was a relatively trivial expense. Burial costs were a tiny proportion of parish expenditure on the poor. In St Martin's overseers spent no more than 2–3 per cent of their total expenditure on burying the parish poor.^80^ Smallpox accounted for just 3.8 per cent of all pauper burials and only 8.7 per cent of pauper children aged under 10. On balance then it appears that the costs of the type of regular inoculations required to control smallpox were probably large compared with the costs of dealing with naturally acquired infections. Vaccination was not necessarily cheaper; however, it did carry the significant advantage that it could be used safely in contexts such as the workhouse without the risk of engendering an outbreak.

Evidence for parochial programmes of inoculation in London is limited. Although the Foundling Hospital is sometimes credited with inoculating all their charges, in fact this policy seems to have been limited to inoculating those older children who had not already been naturally infected upon return to the hospital from rural nursing, or in later periods before they returned, suggesting that the main motive was to prevent epidemics in the hospital itself, as well perhaps as to enhance the children's employment prospects.^81^ Smallpox accounted for 19 per cent of named causes of deaths of Foundling Hospital children aged one to four years nursed in or near London and 18 per cent among those sent to Ackworth in Yorkshire (almost all aged over six).^82^ These data suggest that inoculation was generally too late to reduce the risk of smallpox for this group of foundlings.

The extent of inoculation is very difficult to judge, but it seems clear that both charitable and private inoculation grew in popularity over the last quarter of the eighteenth century in British cities. Razzell's work provides convincing anecdotal evidence of this process in London. However, the apparently slow but undeniable progress of inoculation in large towns and cities after 1760 is in contrast to the abrupt changes in the age structure and levels of smallpox mortality *c.* 1770 evident in data presented here for London and Manchester, and the apparent subsequent stability of these parameters *c.* 1775–1800. It is very hard to discern any progressive effect of inoculation on trends in smallpox mortality over this period despite the documentary evidence of expanding efforts to promote inoculation especially among the urban poor. May has argued that in urban areas the main driver of inoculation programmes was the philanthropic concerns of non‐conformists in particular, and that this was inadequate to create mass programmes of inoculation.^83^ Results presented here would seem to confirm the inadequacy of such private measures against smallpox in large urban populations.

## Footnotes

1
Mercer, *Disease, mortality and population*, ch. 3.

2
Razzell, *Conquest of smallpox*, ch. 1.

3
Jenner, *Inquiry*.

4
Galley and Shelton, ‘Bridging the gap’.

5
Landers, *Death and the metropolis*, pp. 152–7; Davenport, Schwarz, and Boulton, ‘Decline of adult smallpox’.

6
Creighton, *History*, pp. 504–11; Razzell, *Conquest of smallpox*, chs. 3–5; Brunton, ‘Pox Britannica’, pp. 148–72; Smith, *Speckled monster*, pp. 50–63; McNeil, *Plagues and peoples*, pp. 249–50; May, ‘Inoculating the urban poor’; but see Bennett, ‘Inoculation’, for a speculative argument for unobserved but widespread inoculation among the labouring poor in London. Inoculation appears to have been most popular in villages and towns in *southern* England, and the reasons given for its relative failure in large towns have also been used to explain its apparent neglect in the more dispersed rural settlements of northern England and Scotland.

7
Razzell, *Conquest of smallpox*, ch. 5; Smith, *Speckled monster*, ch. 3.

8
Brunton, ‘Pox Britannica’, pp. 157–63; Rusnock, *Vital accounts*, ch. 4.

9
Brunton, ‘Pox Britannica’, pp. 137–40; Razzell, *Conquest of smallpox*, pp. 65–8; Smith, *Speckled monster*, ch. 3.

10 To the extent that the motivation of the London Smallpox Hospital was to isolate victims and prevent spread, the cost of such measures was borne by subscribers, rather than parishes.

11
Haygarth, *Sketch of a plan*, p. 186; Razzell, *Conquest of smallpox*, pp. 72–4; Bennett, ‘Inoculation’, pp. 215–16.

12
Brunton, ‘Pox Britannica’, pp. 169–70.

13
Creighton, *History*, pp. 582–86; Brunton, ‘Pox Britannica’, chs. 6–7. This enthusiasm for vaccination undermines the claim of parental fatalism.

14
Brunton, ‘Pox Britannica’, chs. 4–5; Rusnock, *Vital accounts*, ch. 4; Razzell, ‘Decline’.

15
Davenport et al., ‘Decline of adult smallpox’.

16 The statistical evidence is occasionally fleshed out by anecdotal examples from settlement records, as in the case of Winifred Morris, aged 17 years, who ‘saith she never was married nor bound an apprentice, that she was born at Aberistwyth [sic] in Cardiganshire from whence she came to London about five months ago and was a yearly hired servant to one Mr Maine, a Glover in Panton Street in the Parish of St Martin in the Fields in whose service she was taken ill of the smallpox’. Winifred was admitted to the Workhouse with ‘small pox’; City of Westminster Archive Centre, London (hereafter CWAC), F5031/204, St Martin in the Fields workhouse admissions and discharge registers, 30 Aug. 1739.

17
Razzell, ‘Decline’.

18
Razzell, *Conquest of smallpox*, p. 94.

19
Davenport et al., ‘Decline of adult smallpox’, pp. 1301–3, 1312–13.

20 Razzell suggested that the problem of changes in the recording of infant age evident in the Whitechapel data was also the cause of the increase in infant smallpox mortality we reported in St Martin's. However, we tested for and discounted this possibility, and the possibility of changes in the age structure of the underlying population, by comparing all‐cause and smallpox mortality in infancy and also by comparing the age structure of measles and smallpox burials. Razzell was unable to do this for Whitechapel because he abstracted only the smallpox burials from the burial register.

21
Razzell, ‘Decline’, p. 1333.

22
Razzell, *Conquest of smallpox*, pp. x–xvi. Other evidence supporting this thesis is presented in Brunton, ‘Pox Britannica’, chs. 5 and 7; and Davenport et al., ‘Decline of adult smallpox’, pp. 1298–9.

23
Razzell, *Conquest of smallpox*, pp. xi–xii.

24
Sköld, *Two faces*, p. 166. Razzell, ‘Decline’, p. 1319, cited age distributions of smallpox in Sweden from 1776–1805 to argue that there was no change in the age structure of smallpox mortality in this period and no rise in infectiousness. However, these data cannot address the question of whether there was a shift in the age structure of smallpox burials *c*. 1770 because they only cover the period *after* the change appears to have occurred in Britain: see section III.

25
Percival, ‘Smallpox and measles’, p. 69.

26
Davenport et al., ‘Decline of adult smallpox’, p. 1297.

27
Percival, ‘Smallpox and measles’, pp. 69–70, refers to printed annual Bills of Mortality for Manchester, but the Bills for these years do not appear to have survived. His figures for smallpox burials were derived from the collegiate church sextons' registers which formed the sole basis for the printed Bills in this period.

28
Vigier, *Change and apathy*, pp. 91–9.

29
Chaloner, ‘Manchester’, p. 41, cites a figure of 17,101 for 1757; Percival, ‘Observations’, p. 2, gives a figure of 22,481 for Manchester town in 1774 (analysis of his source gives a count for the town*ship* of 23,966: Chetham's Library, Manchester, Mun.A.4.54–56, census of the town and parish of Manchester 1773–74, enumeration of the houses and inhabitants made by Thomas Percival and John Whittaker); the census of 1801 gave a figure of 70,409 for Manchester township (*Abstract of the Answers*, vol. 1, p. 173).

30 From 1753, when the registers record age and cause of death, the sextons' registers survive for the periods 1 Jan. 1753–31 May 1761; 1 April 1772–18 May 1778; 1 Jan. 1785–Dec. 1811; and Jan. 1815–Dec. 1848. After 31 Dec. 1791 the recording of cause of death and age is incomplete although usable for the period 1 Jan. 1803–31 Dec. 1807 and 1 Jan. 1815–31 Dec. 1820 (the latter period was too late for our purposes).

31 We are grateful to Michael Powell, the archivist of Manchester Cathedral, for allowing us to use the sextons' registers. The registers are described in detail in Powell and Hunwick, ‘Manchester cathedral’.

32 We are grateful to John Marsden and the Manchester and Lancashire Family History Society for providing a machine‐readable version of their transcription of parish register burials in Manchester. The St John Deansgate burial register included 10,685 burials in the period 1769–1812. Of these only 25 lacked cause of death and 25 lacked exact age.

33 Adults aged 10–39 comprised 17.8% of burials in St Martin's in 1752–66 and 20.2% in Manchester in 1753–61; 16.6% in St Martin's in 1775–99 and 16.7% in Manchester in 1772–91. We have no direct census‐type records of the population age structure of St Martin in the Fields or Manchester before 1841. However, a ‘census’ of Manchester in 1774 that listed inhabitants in three age groups indicated that the age structure of Manchester was typical of many towns in this period in its female‐biased adult sex ratio and preponderance of young adults, and very similar to the age structure of Westminster (of which St Martin's formed 16%) in 1821; Percival, ‘Observations’, p. 2.

34
Creighton, *History*, p. 533.

35 Although the number of adult smallpox burials was small in each period the percentage of adult smallpox burials in the first period was significantly different from each of the later periods (*p* < 0.01 by two‐tailed z‐tests).

36
Davenport et al., ‘Decline of adult smallpox’, tab. 2, p. 1300.

37
Ibid., pp. 1300–1.

38 The percentage depends on whether the few burials where sex was not clear were all assumed (in the extreme case) to be female, or were distributed equally by sex.

39 Occupations were not generally recorded in the collegiate church sextons' books, but burials of soldiers were noted.

40
Landers, *Death and the metropolis*, pp. 286–99.

41 Wrigley's widely used estimate for London: Wrigley, ‘Simple model’, p. 44.

42 See Davenport et al., ‘Decline of adult smallpox’, nn. 77, 86, and section III below for discussions of the persistence of epidemic cycles superimposed on a persistent background of weekly or monthly smallpox burials.

43
Cunningham, ‘Married at the Coll.’.

44
Davenport et al., ‘Decline of adult smallpox’, p. 1304.

45 Compared with less than 20% of burials aged one in St Martin's in the same period (tab. 4). While the difference in infancy could reflect greater under‐recording of neonatal burials in Manchester than London (ages where mortality rates are highest and where smallpox is usually a trivial cause of death), it is unlikely that any similar bias in recording of burials by age affected older child ages.

46
Percival, ‘Observations’, p. 6.

47 See Wrigley, Davies, Oeppen, and Schofield, *Population history*, pp. 12–17, for a description of the technique, and Newton, ‘Family reconstitution’, for its application to urban populations.

48
Finlay, *Population and metropolis*; Landers, *Death and the metropolis*, chs. 4–5; Galley, *Demography*, chs. 1, 3, 6–7; Newton, ‘Family reconstitution’.

49 The procedures used for family reconstitution and estimates of mortality rates are documented by Davenport, Boulton, and Black, ‘Origins’. For a detailed study of the traffic in corpses in St Martin's, see Boulton, ‘Traffic in corpses’.

50 These rates were calculated using multiple decrement product‐limit life tables that took into account changes in the population at risk throughout each age interval. Smallpox‐specific rates were calculated on the assumption that the risk of dying from smallpox was independent of the risks of exiting the sample for other reasons (death from another cause or removal of the family from observation).

51 The sample was representative of the parish as a whole with respect to the distributions of burial and baptism fees, except that it excluded unmarried women and illegitimate children, most of whom were delivered in the parish workhouse. Illegitimate infants comprised a low and fairly constant proportion of baptisms in the parish (*c*. 3%). Although the proportions paying for public and private baptisms changed between the third and fourth quarters of the eighteenth century, the proportions of the reconstitution sample and the parish that were pauper or very expensive baptisms remained fairly constant (roughly 10% each). Although classification of families by baptism fee paid permitted the estimation of mortality rates by social status groups, the numbers of smallpox burials were too low to allow calculation of age‐specific smallpox mortality rates by social status.

52
Landers, *Death and the metropolis*, pp. 154–6.

53
Anderson and May, *Infectious diseases*, chs. 4–6; Duncan, Scott, and Duncan, ‘Dynamics of smallpox epidemics’.

54
Anderson and May, *Infectious diseases*, pp. 128–43; Duncan et al., ‘Dynamics of smallpox epidemics’.

55
Duncan, Duncan, and Scott, ‘Oscillatory dynamics’.

56
Krylova, ‘Predicting epidemiological transitions’.

57
Ibid., pp. 103–6.

58
Ibid., p. 170.

59
Carmichael and Silverstein, ‘Smallpox in Europe’; Creighton, *History*, pp. 434–58. Note that the long‐run pattern of smallpox mortality in Geneva, fig. 3, lends no weight to this argument.

60
Perrenoud, ‘Contribution’, p. 183. The impact of inoculation is unclear. Wenger, ‘ “De petites considérations” ’, argued that inoculation was popular among wealthy Genevoise from the 1750s but charitable provision of inoculation to the poor failed.

61
Perrenoud, ‘Contribution’, p. 186.

62
Ibid., p. 195.

63
Davenport et al., ‘Decline of adult smallpox’, p. 1309.

64 For acute infections transmitted directly from human to human, there are in fact evolutionary trade‐offs between infectiousness and virulence, since higher virulence may be associated with lower host mobility and/or avoidance by other potential hosts, factors which limit transmission. The high virulence of smallpox was associated with relatively low infectiousness, but also with the capacity to persist for long periods outside a human host, a feature that permitted sustained transmission despite the lethal and obvious nature of the disease; Walther and Ewald, ‘Pathogen survival’.

65
Fridlizius, ‘Mortality decline’.

66 Calculated from data in Vaccination Commission, *First Report* (P.P. 1889, XXXIX), app., pp. 107–8.

67
Johansen, *Danish population history*, p. 86. Johansen also argued (ibid., p. 109) that inoculation was limited to Copenhagen but the fall in smallpox mortality was general for the country as a whole.

68
Sköld, *Two faces*, ch. 4.

69
Sköld, *Two faces*, ch. 4; Fridlizius, ‘Mortality decline’.

70
Pitkanen, Mielke, and Jorde, ‘Smallpox and its eradication’, p. 98. Measles mortality was estimated and subtracted from these measures for the period 1751–73.

71
Fenner, Henderson, Arita, Ježek, and Ladnyi, *Smallpox and its eradication*, ch. 4.

72
Li, Carroll, Gardner, Walsh, Vitalis, and Damon, ‘Origins of smallpox’.

73
Fenner et al., *Smallpox and its eradication*, ch. 8.

74
Creighton, *History*, pp. 544–52.

75
Razzell, ‘Decline’, pp. 1318–20.

76 CWAC, F625 (unpaginated), ‘Expences to Teddington to Inoculate Children’ cost £3 15s. 1d.

77 CWAC, F2076/286. For orders to vaccinate on pain of expulsion, see ibid., 342, and F2077, 21 Jan. 1817.

78 CWAC, F2006/454.

79 CWAC, F462/154–328.

80 This has been calculated from the overseers' accounts (CWAC, F485, F488, F492, F495) for the years 1733–6, and workhouse accounts (CWAC, F491, F2212) for the same period.

81
Miller, *Adoption of inoculation*, pp. 144–5; Bennett, ‘Inoculation’, pp. 205, 215; Creighton, *History*, pp. 514–15.

82
Levene, *Childcare*, pp. 160–2.

83
May, ‘Inoculating the urban poor’.
